# Supplementary material for: Depressive symptoms and association with prevalent cardiovascular disease: A cross-sectional analysis in Port-au-Prince, Haiti
Source: PLOS Ment Health. 2026 Aug 27;3(8):e0000693. doi: 10.1371/journal.pmen.0000693 (PMC13521383; doi:10.1371/journal.pmen.0000693)
Supplement: Supplementary file 3 [file pmen.0000693.s004.docx]

Thank you for the opportunity to revise and resubmit our manuscript. Below we have **bolded** our responses to all points raised by the editor and reviewers. We have uploaded a track change and cleaned version of the manuscript into the editorial system.

**Editor**

Overview: Thank you for submitting your manuscript to PLOS Mental Health. I am very sorry for the delay. After careful consideration of the reviewer reports, which we have now received, we feel that your paper has merit but does not fully meet PLOS Mental Health’s publication criteria as it currently stands. Therefore, we invite you to submit a revised version of the manuscript that addresses the points raised during the review process.

**Thank you for the positive comment and we have shared a revised version of the manuscript that addressed all the points raised during the review process.**

1. Please include a complete copy of PLOS’ questionnaire on inclusivity in global research in your revised manuscript. Our policy for research in this area aims to improve transparency in the reporting of research performed outside of researchers’ own country or community. The policy applies to researchers who have travelled to a different country to conduct research, research with Indigenous populations or their lands, and research on cultural artefacts. The questionnaire can also be requested at the journal’s discretion for any other submissions, even if these conditions are not met. Please find more information on the policy and a link to download a blank copy of the questionnaire here: https://journals.plos.org/mentalhealth/s/best-practices-in-research-reporting. Please upload a completed version of your questionnaire as Supporting Information when you resubmit your manuscript.

**We uploaded a completed version of the questionnaire attached with the manuscript**. **And referenced it in the method section, subtitle “Ethics Statement” line 128-130**

2. For studies involving third-party data, we encourage authors to share any data specific to their analyses that they can legally distribute. PLOS recognizes, however, that authors may be using third-party data they do not have the rights to share. When third-party data cannot be publicly shared, authors must provide all information necessary for interested researchers to apply to gain access to the data. (https://journals.plos.org/plosone/s/data-availability#loc-acceptable-data-access-restrictions)

For any third-party data that the authors cannot legally distribute, they should include the following information in their Data Availability Statement upon submission:

1) A description of the data set and the third-party source

2) If applicable, verification of permission to use the data set

3) Confirmation of whether the authors received any special privileges in accessing the data that other researchers would not have

4) All necessary contact information others would need to apply to gain access to the data

**N/A**

3. Please upload separate figure files in .tif or .eps format. Also, remove the figures from your manuscript file but keep the legends.

**We have removed the figures from the manuscript file and uploaded them in eps format**

4. Please ensure that your Ethics Statement is available in its entirety at the beginning of your Methods section, under a subheading 'Ethics Statement'.

**Ethics statement was moved at the beginning of the methods section, line 124-130**

5. We notice that your supplementary figures/tables are included in the manuscript file. Please remove them and upload them with the file type 'Supporting Information'. Please ensure that each Supporting Information file has a legend listed in the manuscript after the references list.

**We have removed the supplementary figures and tables in the manuscript. We have uploaded them with the file Supporting Information and kept their legend in the manuscript.**

**Reviewer 1**

Overview: This is a cross-sectional analysis of the association between depression and baseline presence of CVD (defined as angina, MI, TIA, stroke, HF) in Haiti. No prior research from Haiti on this topic is available, and as a low-income country with numerous social stressors, this is a worthwhile study topic. The cohort from which the data are drawn is a major strength with rigorous data collection and even adjudication of prevalent CVD, which is unusual and adds rigor. The finding that depressive symptoms are associated among women, but not significantly among men, is intriguing and worth reporting.

**Thank you for the positive comment**

Major comments:

1. An important issue needs to be addressed in this report relating to the odd handling of interactions. Multiplicative interactions are appropriate for proportions, as in time-to-event analyses that use proportional hazards. This is a cross-sectional study where additive interactions are more appropriate, since the aim here is to assess risk differences. The language about stratifying because the interaction was not significant should be removed as it is incorrect. The language in the statistical appendix is consistent with usual statistical practice and should be reflected in the main text analysis plan, that is, there was evidence of effect modification in the interaction test, therefore analyses were stratified.

**We agree that assessing interactions on the additive scale is more useful than on the multiplicative scale, especially when it comes to identifying groups to prioritize resources, such as by sex here. Given the cross-sectional design, we cannot directly calculate risk differences, but with prevalence ratios approximating risk ratios, we were able to quantify additive effects using the relative excess risk index (RERI) based on Rothman et al^*^. We found an additive effect of being both Moderate-severe DS and being female (RERI=0.07) on CVD prevalence. To make this clearer for readers as well, we also provided the prevalence ratios in Table 4 as well as now including the prevalence of CVD by each group (Moderate-severe DS and female, Moderate-severe DS and male, not Moderate-severe DS and female, not Moderate-severe DS and male).**

**We also agree that the interaction term in the Poisson regression is greater than 0.05 and we can’t acknowledge effect modification on the multiplicative scale. We have edited the results section accordingly line 265-268 and the discussion section line 343-346**

**The language about stratifying was removed as suggested.**

*** Lash TL, VanderWeele TJ, Haneuse S, Rothman KJ. Modern Epidemiology, Chapter 26 Analysis of Interaction: Wolters Kluwer; 2021.**

2. Given the presence of effect modification, the discussion of sex differences is quite appropriate and could even be expanded. There are two references to prior findings of stronger associations in women from Asia. Expanding these findings could strengthen this report and its contribution. Were these also low-income countries.

**Thank you for this helpful suggestion. We have expanded the discussion section to further contextualize prior studies from Asia that reported stronger associations between depressive symptoms and cardiovascular disease among women. Specifically, we now note that these studies were conducted in Japan and China, which are classified as high-income and upper-middle-income countries, respectively. However, the Chinese cohort was drawn from Guizhou Province, one of the country’s least economically developed regions, where socioeconomic adversity and limited healthcare access may share contextual similarities with Haiti. These additions have been incorporated into the revised discussion section line 319-325.**

Minor details that need attention:

3. Technically the PHQ is a screening tool, thus language should be modified to refer to ‘moderate/severe depressive symptoms’ rather than ‘depression.’

**This is a good point. We have clarified the terminology throughout the manuscript to refer to moderate to severe depressive symptoms rather than depression, consistent with the PHQ-9 as a screening instrument.**

4. Given that this tool has not been validated in Haiti, it might be useful to report the Crohnbach’s alpha and comment on its similarity to the US where this instrument was developed.

**Thank you for this helpful suggestion. We also reported the internal consistency of the PHQ-9 in our sample (Cronbach’s alpha = 0.78) from line 155-159, indicating good reliability. To provide context, we added a comparison to reliability estimates reported in U.S. populations, where Cronbach’s alpha values typically range from approximately 0.79‒0.93 across diverse clinical and community settings. These revisions have been incorporated into the Methods section with appropriate references:**

- **Palmer, E. O. C., Ker, S., Rentería, M. E., Carmody, T., & Rush, A. J. (2024). Psychometric evaluation and linking of the PHQ-9, QIDS-C, and VQIDS-C in a real-world population with major depressive disorder. *Neuropsychiatr Dis Treat*, *20*, 671-687. https://doi.org/10.2147/ndt.S444223**
- **Huang, F. Y., Chung, H., Kroenke, K., Delucchi, K. L., & Spitzer, R. L. (2006). Using the Patient Health Questionnaire-9 to measure depression among racially and ethnically diverse primary care patients. *J Gen Intern Med*, *21*(6), 547-552. https://doi.org/10.1111/j.1525-1497.2006.00409.x**
- **Patel, J. S., Oh, Y., Rand, K. L., Wu, W., Cyders, M. A., Kroenke, K., & Stewart, J. C. (2019). Measurement invariance of the patient health questionnaire-9 (PHQ-9) depression screener in U.S. adults across sex, race/ethnicity, and education level: NHANES 2005-2016. *Depress Anxiety*, *36*(9), 813-823. https://doi.org/10.1002/da.22940**
- **Shaff, J., Kahn, G., & Wilcox, H. C. (2023). An examination of the psychometric properties of the Patient Health Questionnaire-9 (PHQ-9) in a Multiracial/ethnic population in the United States. *Front Psychiatry*, *14*, 1290736. https://doi.org/10.3389/fpsyt.2023.1290736**

5. Please provide the prevalence of CVD in the mod-severe depressive symptom group vs. non depressive symptom group in the abstract to give context for interpreting the PR.

**Thank you for this helpful suggestion, which aids in the interpretation of the prevalence ratios. We have added the prevalence of cardiovascular disease by depressive symptom severity to the Results section of the abstract, line 63‒65**

**“The prevalence of CVD was higher among participants with moderate–severe DS compared with those with none–mild DS, with similar patterns observed in both sexes (males: 21.5% vs 10.6%; females: 23.3% vs 15.3%).”**

6. Line 108 – this is discussing a prevalence, not a rate.

**We reframed this terminology as suggested.**

7. Why is there a heading “CVD outcome” in the methods? There are no outcomes in this cross-sectional study. Perhaps retitle this “CVD” or “Dependent variable: CVD”.

**Thank you, the title was edited as suggested, line 162**

8. It is unusual to adjudicate the baseline prevalence of disease. Could comment on why this was done.

**In high income countries, baseline prevalence of CVD in CVD Cohorts (for example in the US: MESA, REGARDS, CARDIA, JHS, etc) is usually not adjudicated and only determined based on patient reported diagnosis, given relatively robust healthcare infrastructure, systems, and a higher level of healthcare accessibility. However, in Haiti as well as many low-income countries, patients do not have reliable access to healthcare due to severe poverty, fragile health systems, etc and many patients with chronic illnesses are undiagnosed. Therefore, we adjudicated baseline prevalence given many cases of prevalent disease would be missed if we relied only on patient self-report.**

9. Please spell out REGARDS and CARDIA first time used.

**We have spelled out REGARDS and CARDIA as suggested, line 165‒167**

**“REasons for Geographic and Racial Differences in Stroke (REGARDS) and Coronary Artery Risk Development in Young Adults Study (CARDIA)”**

10. Please provide references for the mediating role of HTN in depression (line 188-189).

**We have added references to support the conceptual placement of hypertension as a potential mediator variable in the causal pathway between DS and CVD. Prior studies have demonstrated associations between DS or depression and incident hypertension and the contribution of hypertension to cardiovascular risk, although the extent of mediation remains uncertain. Relevant references have now been cited in the Methods section, line 195.**

**References # 28, 35, 36 respectively:**

- **Levine GN, Cohen BE, Commodore-Mensah Y, et al. Psychological Health, Well-Being, and the Mind-Heart-Body Connection: A Scientific Statement From the American Heart Association. Circulation. 2021;143(10):e763-e783. doi:doi:10.1161/CIR.0000000000000947**
- **Obas KA, Kwiatkowski M, Schaffner E, et al. Depression and cardiovascular disease are not linked by high blood pressure: findings from the SAPALDIA cohort. Sci Rep. Apr 1 2022;12(1):5516. doi:10.1038/s41598-022-09396-2**
- **Tu Q, Lin S, Hafiz N, et al. Independent and joint associations of hypertension and depression with cardiovascular diseases and all-cause mortality: a population-based cohort study. J Hum Hypertens. Sep 2025;39(9):634-642. doi:10.1038/s41371-025-01045-1**

11. Table 3. Percents are not correct – among the none/mild depressive sx, 10.6% had CVD (122+1028 =1150, 122/1150 = 10.6%). Prevalence of CVD among those with mod/severe sx was 21.5%, not 27.4% (23+84 = 107, 23/107 = 21.50%).

**Thank you for carefully reviewing Table 3 and identifying this error due to a formatting error in the presentation of descriptive statistics. We have recalculated all percentages using the correct denominators (number with CVD divided by the total number within each depression severity category).**

**The corrected prevalence estimates are:**

- **10.6% among male participants with none to mild depressive symptoms and 21.5% among those with moderate to severe symptoms**
- **15.3% among female participants with none to mild depressive symptoms and 23.3% among those with moderate to severe symptoms.**

**We have updated Table 3 accordingly.**

12. Consider making Table S2 main findings – the graded relationship adds strength to the inferences.

**Table S2 was renamed to Table 3 and findings described in the results section are now linked to the new Table 3.**

13. Not sure why the sensitivity analysis is referred to as “post hoc”. This is usually used in clinical trials when analyses are done after the data have been analyzed and those analyses were not planned in designing the trial. This is a cross-sectional observational study, not a trial.

**The language was corrected as suggested.**

**REVIEWER #2**

1. The current Data Availability Statement indicates that only a subset of de-identified data may be shared upon request, a signed data access agreement, and only beginning three years after publication. This is not aligned with PLOS’s stated expectation that data necessary to replicate findings be made publicly available without restriction at the time of publication, with only rare exceptions.

**The dataset has been assigned the reserved DOI: https://doi.org/10.17632/5bmc66w395, and public release of the repository record will be synchronized with publication of the manuscript. Following publication, qualified researchers may request access to the de-identified IPD through the repository landing page associated with the dataset DOI, subject to applicable data use requirements.**

**We have reviewed the Data Availability Statement:**

**“De-identified individual participant data underlying the findings reported in this manuscript have been deposited in Digital Commons Data@Becker and made available through controlled access under a Data Use Agreement (https://doi.org/10.17632/5bmc66w395). To protect participant confidentiality, only aggregated summary data will be shared openly.”**

2. I encourage revision because the setting, sample size, and focus on depression as a potential cardiovascular correlate are valuable.

**Thank you for the positive comment**

3. Outcome definition and adjudication are insufficiently reproducible and may be differentially misclassified. The authors define prevalent CVD as a composite of angina, MI, TIA/stroke, and HF and state that outcomes were adjudicated using epidemiologic criteria aligned with AHA/WHO/ESC. However, the manuscript does not provide the operational algorithm for each component, what proportion relied on self-report vs objective testing, who adjudicated and whether adjudicators were blinded to PHQ-9, and any reliability metrics. This is critical because depression can influence symptom reporting (particularly chest pain/angina-like symptoms), creating differential misclassification and inflating associations. So, please Provide a detailed adjudication appendix, including explicit criteria for each CVD component. Report prevalence of each CVD component separately and run component-specific analyses.

**Thank you for this comment. Detailed adjudication criteria for heart failure, stroke/TIA, myocardial infarction, and angina were previously developed and published for the Haiti Cardiovascular Disease Cohort. To improve reproducibility, we have now added (Supporting information S1 Appendix Table 1) summarizing the adjudication algorithms used for each CVD component and expanded the Methods section accordingly. These criteria incorporated participant-reported symptoms, physical examination findings, imaging results, laboratory data, and physician adjudication by a three-member clinical committee. We have also reported the prevalence of each CVD component separately (Supporting information S1 Appendix Table 2).**

**References #2**

**Yan LD, Sufra R, St Sauveur R, et al. Spectrum of prevalent cardiovascular diseases in urban Port-au-Prince, Haiti: a population-based cross-sectional study. Lancet Reg Health Am. May 2024;33:100729. doi:10.1016/j.lana.2024.100729**

4. The authors state confounders were selected via a DAG and adjusted for socioeconomic factors and lifestyle factors (smoking, alcohol, physical activity, BMI) and stress. However, several of these variables can be mediators of depression’s effect on CVD (e.g., depression → reduced activity → obesity → CVD), so adjusting for them estimates something closer to a controlled direct effect, not the total association. Conversely, key traditional CVD confounders (diabetes, dyslipidemia, CKD, family history) are not mentioned.

**We agree that, in a cross-sectional analysis, variables such as physical activity and BMI may function as either confounders or mediators in the relationship between depressive symptoms (DS) and cardiovascular disease (CVD). In our primary analysis, these variables were included based on the hypothesized causal structure represented in the DAG. However, given this uncertainty, we also conducted a sensitivity analysis excluding physical activity and BMI from the adjusted model. The association was slightly stronger but substantively unchanged (aPR = 1.48; 95% CI: 1.18–1.85) compared with the primary analysis (aPR = 1.36; 95% CI: 1.08–1.72) (Supporting information, Table S2). These findings have been referenced in the discussion section line 296-298 and the results section line 273-276**

**Traditional cardiovascular risk factors such as hypertension, diabetes, and dyslipidemia are explicitly considered during DAG development (Fig. S2, CV rf) and we conceptualized them as potential mediators linking DS to CVD rather than primary confounders. Therefore, they were not included in the primary adjustment set to avoid potential overadjustment.**

**We have revised the Methods section (lines 180–185) to clarify that the since the primary model conditions on physical activity and BMI, it may estimate something closer to a controlled direct association rather than the total causal effect. We also provide additional justification for the selected adjustment set.**

**Finally, we acknowledge that some traditional cardiovascular risk factors may act as confounders in certain settings. Residual confounding from incompletely measured or unavailable cardiovascular risk factors cannot be excluded, and this limitation has been acknowledged in the revised manuscript.**

5. The fully adjusted model drops 98 observations (N=2897) due to missingness. The authors do not describe missingness mechanisms, whether missingness differs by depression or CVD, nor any sensitivity analysis (e.g., multiple imputation). For a mental health exposure strongly correlated with socioeconomic adversity, complete-case analysis can bias estimates.

**Thank you for this important methodological observation. The fully adjusted model excluded 98 participants (3.3%) due to missing covariate data. Among the recruited cohort, the outcome variable (CVD) had no missingness, while depressive symptoms had minimal missingness (n = 10). Missingness in covariates ranged from 5 to 64 observations depending on the variable. To address this concern, under the MAR assumption, we conducted a sensitivity analysis using multiple imputation by chained equations (MICE; R package *mice* v3.19.0), generating 20 imputed datasets with estimates pooled according to Rubin’s rules. We note that selection into the cohort may result in missing data and this may be caused by depression and/or CVD, and this can bias our results and this is acknowledged as a limitation in the Discussion section. (line 360-364)**

**Of 3,005 participants, 98 (3.3%) had been excluded from the complete-case analysis. Crucially, pooled estimates from the imputed datasets were virtually identical to our original complete-case results. The association between moderate-to-severe DS and CVD prevalence remained statistically significant and of nearly identical magnitude (complete-case: aPR 1.36, 95% CI 1.08–1.72; MI-pooled: aPR 1.37, 95% CI 1.09–1.71). (Supplemental Table S2).**

**We have added a paragraph to the Method (line 235-245) and Results section (line 276-279) and a supplemental comparison table to reflect this sensitivity analysis.**

6. The title refers to “Prevalent Heart Disease,” but the outcome includes stroke/TIA.

**We edited the title with Prevalent Cardiovascular Disease, line 1**

Minor revisions:

7. Physical activity frequencies appear swapped between Table 1 and Table S1 (counts by sex in Table S1 sum to the opposite category totals reported in Table 1).

**The subcategories title under “physical activity” were erroneously swapped thank you for this important correction.**

8. Table S2 contains inverted CI presentation for mild depression (e.g., “1.27 (2.02–1.57)”).

**The correction is made; there was a typo in the CI it’s 1.02 – 1.57 thank you for this comment**

9. BMI category ranges in Methods are incorrectly (underweight).

**Thank you we made the correction on the numbers of BMI category.**
