# Supplementary material for: Depressive symptoms and association with prevalent cardiovascular disease: A cross-sectional analysis in Port-au-Prince, Haiti
Source: PLOS Ment Health. 2026 Aug 27;3(8):e0000693. doi: 10.1371/journal.pmen.0000693 (PMC13521383; doi:10.1371/journal.pmen.0000693)
Supplement: Peer review history

# Peer review: Decision Letter 0

**DOI:** 10.1371/journal.pmen.0000693.r001

**Authors:** Karli Montague-Cardoso

6 Apr 2026

PMEN-D-25-00557

Depression and association with Prevalent Heart Disease: A Cross-Sectional Analysis in Port-au-Prince, Haiti

PLOS Mental Health

Dear Dr. Pierre,

Thank you for submitting your manuscript to PLOS Mental Health. I am very sorry for the delay. After careful consideration of the reviewer reports, which we have now received, we feel that your paper has merit but does not fully meet PLOS Mental Health’s publication criteria as it currently stands. Therefore, we invite you to submit a revised version of the manuscript that addresses the points raised during the review process.

Please address all of the comments made, which you can find at the end of this email.

Please submit your revised manuscript by May 20 2026 11:59PM. If you will need more time than this to complete your revisions, please reply to this message or contact the journal office at mentalhealth@plos.org. When you're ready to submit your revision, log on to https://www.editorialmanager.com/pmen/ and select the 'Submissions Needing Revision' folder to locate your manuscript file.

Please include the following items when submitting your revised manuscript:

- A letter that responds to each point raised by the editor and reviewer(s). You should upload this letter as a separate file labeled 'Response to Reviewers'.
- A marked-up copy of your manuscript that highlights changes made to the original version. You should upload this as a separate file labeled 'Revised Manuscript with Track Changes'.
- An unmarked version of your revised paper without tracked changes. You should upload this as a separate file labeled 'Manuscript'.

Guidelines for resubmitting your figure files are available below the reviewer comments at the end of this letter.

As the corresponding author, your ORCID iD is verified in the submission system and will appear in the published article. PLOS supports the use of ORCID, and we encourage all coauthors to register for an ORCID iD and use it as well. Please encourage your coauthors to verify their ORCID iD within the submission system before final acceptance, as unverified ORCID iDs will not appear in the published article. *Only*  the individual author can complete the verification step; PLOS staff *cannot*  verify ORCID iDs on behalf of authors.

We look forward to receiving your revised manuscript.

Kind regards,

Karli Montague-Cardoso

Staff Editor

PLOS Mental Health

**Journal Requirements:**

1. Please include a complete copy of PLOS’ questionnaire on inclusivity in global research in your revised manuscript. Our policy for research in this area aims to improve transparency in the reporting of research performed outside of researchers’ own country or community. The policy applies to researchers who have travelled to a different country to conduct research, research with Indigenous populations or their lands, and research on cultural artefacts. The questionnaire can also be requested at the journal’s discretion for any other submissions, even if these conditions are not met.  Please find more information on the policy and a link to download a blank copy of the questionnaire here: https://journals.plos.org/mentalhealth/s/best-practices-in-research-reporting. Please upload a completed version of your questionnaire as Supporting Information when you resubmit your manuscript.

2. For studies involving third-party data, we encourage authors to share any data specific to their analyses that they can legally distribute. PLOS recognizes, however, that authors may be using third-party data they do not have the rights to share. When third-party data cannot be publicly shared, authors must provide all information necessary for interested researchers to apply to gain access to the data. (https://journals.plos.org/plosone/s/data-availability#loc-acceptable-data-access-restrictions)

For any third-party data that the authors cannot legally distribute, they should include the following information in their Data Availability Statement upon submission:

1) A description of the data set and the third-party source

2) If applicable, verification of permission to use the data set

3) Confirmation of whether the authors received any special privileges in accessing the data that other researchers would not have

4) All necessary contact information others would need to apply to gain access to the data

3. Please upload separate figure files in .tif or .eps format. Also, remove the figures from your manuscript file but keep the legends.

For more information about figure files please see our guidelines:

https://journals.plos.org/mentalhealth/s/figures

https://journals.plos.org/mentalhealth/s/figures#loc-file-requirements

4. Please ensure that your Ethics Statement is available in its entirety at the beginning of your Methods section, under a subheading 'Ethics Statement'.

5. We notice that your supplementary figures/tables are included in the manuscript file. Please remove them and upload them with the file type 'Supporting Information'. Please ensure that each Supporting Information file has a legend listed in the manuscript after the references list.

If the reviewer comments include a recommendation to cite specific previously published works, please review and evaluate these publications to determine whether they are relevant and should be cited. There is no requirement to cite these works unless the editor has indicated otherwise.

**Additional Editor Comments (if provided):**

[Note: HTML markup is below. Please do not edit.]

Reviewers' comments:

Reviewer's Responses to Questions

**Comments to the Author**

1. Does this manuscript meet PLOS Mental Health’s publication criteria?

Reviewer #1: Yes

Reviewer #2: Partly

**********

2. Has the statistical analysis been performed appropriately and rigorously?

Reviewer #1: No

Reviewer #2: Yes

**********

3. Have the authors made all data underlying the findings in their manuscript fully available (please refer to the Data Availability Statement at the start of the manuscript PDF file)?

The PLOS Data policy

Reviewer #1: Yes

Reviewer #2: No

**********

4. Is the manuscript presented in an intelligible fashion and written in standard English?

Reviewer #1: Yes

Reviewer #2: Yes

**********

**Reviewer #1:**  This is a cross-sectional analysis of the association between depression and baseline presence of CVD (defined as angina, MI, TIA, stroke, HF) in Haiti. No prior research from Haiti on this topic is available, and as a low-income country with numerous social stressors, this is a worthwhile study topic. The cohort from which the data are drawn is a major strength with rigorous data collection and even adjudication of prevalent CVD, which is unusual and adds rigor. The finding that depressive symptoms are associated among women, but not significantly among men, is intriguing and worth reporting.

An important issue need to be addressed in this report relating to the odd handling of interactions. Multiplicative interactions are appropriate for proportions, as in time-to-event analyses that use proportional hazards. This is a cross-sectional study where additive interactions are more appropriate, since the aim here is to assess risk differences. Further, p-values for interactions are rarely restrained to 0.05 unless the data set is very large, which this one is not. Many tests for interaction are considered important when p<0.10 or even 0.15. The p=0.07 supports the likelihood of effect modification, therefore the decision to stratify on sex and draw conclusions about observed sex differences is supported. The language about stratifying because the interaction was not significant should be removed as it is incorrect. The language in the statistical appendix is consistent with usual statistical practice, and should be reflected in the main text analysis plan, that is, there was evidence of effect modification in the interaction test, therefore analyses were stratified.

Given the presence of effect modification, the discussion of sex differences is quite appropriate and could even be expanded. There are two references to prior findings of stronger associations in women from Asia. Expanding on these findings could strengthen this report and its contribution. Were these also low-income countries? Hopefully a follow-on study will come soon from this cohort to see if the findings are also observed in the longitudinal context with incident CVD.

Minor details that need attention:

Technically the PHQ is a screening tool, thus language should be modified to refer to ‘moderate/severe depressive symptoms’ rather than ‘depression.’

Given that this tool has not been validated in Haiti, it might be useful to report the Crohnbach’s alpha and comment on its similarity to the US where this instrument was developed.

Please provide the prevalence of CVD in the mod-severe depressive symptom group vs. non depressive symptom group in the abstract to give context for interpreting the PR.

Line 108 – this is discussing a prevalence, not a rate.

Why is there a heading “CVD outcome” in the methods? There are no outcomes in this cross-sectional study. Perhaps retitle this “CVD” or “Dependent variable: CVD”.

It is unusual to adjudicate the baseline prevalence of disease. Could comment on why this was done.

Please spell out REGARDS and CARDIA first time used.

Please provide references for the mediating role of HTN in depression (line 188-189).

Table 3. Percents are not correct – among the none/mild depressive sx, 10.6% had CVD (122+1028 =1150, 122/1150 = 10.6%). Prevalence of CVD among those with mod/severe sx was 21.5%, not 27.4% (23+84 = 107, 23/107 = 21.50%).

Consider making Table S2 main findings – the graded relationship adds strength to the inferences.

Not sure why the sensitivity analysis is referred to as “post hoc”. This is usually used in clinical trials when analyses are done after the data have been analyzed and those analyses were not planned in designing the trial. This is a cross-sectional observational study, not a trial.

**Reviewer #2:**  1. The current Data Availability Statement indicates that only a subset of de-identified data may be shared upon request, a signed data access agreement, and only beginning three years after publication. This is not aligned with PLOS’s stated expectation that data necessary to replicate findings be made publicly available without restriction at the time of publication, with only rare exceptions.

2. I encourage revision because the setting, sample size, and focus on depression as a potential cardiovascular correlate are valuable. Below I list major issues that must be addressed:

- Outcome definition and adjudication are insufficiently reproducible and may be differentially misclassified. The authors define prevalent CVD as a composite of angina, MI, TIA/stroke, and HF and state that outcomes were adjudicated using epidemiologic criteria aligned with AHA/WHO/ESC. However, the manuscript does not provide the operational algorithm for each component, what proportion relied on self-report vs objective testing, who adjudicated and whether adjudicators were blinded to PHQ-9, and any reliability metrics. This is critical because depression can influence symptom reporting (particularly chest pain/angina-like symptoms), creating differential misclassification and inflating associations. So, please Provide a detailed adjudication appendix, including explicit criteria for each CVD component. Report prevalence of each CVD component separately and run component-specific analyses.

- The authors state confounders were selected via a DAG and adjusted for socioeconomic factors and lifestyle factors (smoking, alcohol, physical activity, BMI) and stress. However, several of these variables can be mediators of depression’s effect on CVD (e.g., depression → reduced activity → obesity → CVD), so adjusting for them estimates something closer to a controlled direct effect, not the total association. Conversely, key traditional CVD confounders (diabetes, dyslipidemia, CKD, family history) are not mentioned.

- The fully adjusted model drops 98 observations (N=2897) due to missingness. The authors do not describe missingness mechanisms, whether missingness differs by depression or CVD, nor any sensitivity analysis (e.g., multiple imputation). For a mental health exposure strongly correlated with socioeconomic adversity, complete-case analysis can bias estimates.

- The title refers to “Prevalent Heart Disease,” but the outcome includes stroke/TIA.

-

Minor revisions:

- Physical activity frequencies appear swapped between Table 1 and Table S1 (counts by sex in Table S1 sum to the opposite category totals reported in Table 1).

- Table S2 contains inverted CI presentation for mild depression (e.g., “1.27 (2.02–1.57)”).

- BMI category ranges in Methods are incorrectly (underweight).

**********

what does this mean?). If published, this will include your full peer review and any attached files.

**Do you want your identity to be public for this peer review?**  If you choose “no”, your identity will remain anonymous but your review may still be made public.

For information about this choice, including consent withdrawal, please see our Privacy Policy

Reviewer #1: No

Reviewer #2: **Yes:**  An Viet Tran

**********

[NOTE: If reviewer comments were submitted as an attachment file, they will be attached to this email and accessible via the submission site. Please log into your account, locate the manuscript record, and check for the action link "View Attachments". If this link does not appear, there are no attachment files.]

**Figure Resubmissions:**

# Author comment: Author response to Decision Letter 1

**DOI:** 10.1371/journal.pmen.0000693.r002

30 Jun 2026

Attachment Submitted filename: Response_reviewers.docx

# Editor report: Decision Letter 1

**DOI:** 10.1371/journal.pmen.0000693.r003

**Authors:** Karli Montague-Cardoso

29 Jul 2026

Depressive Symptoms and Association with Prevalent Cardiovascular Disease: A Cross-Sectional Analysis in Port-au-Prince, Haiti

PMEN-D-25-00557R1

Dear Dr Pierre,

We are pleased to inform you that your manuscript 'Depressive Symptoms and Association with Prevalent Cardiovascular Disease: A Cross-Sectional Analysis in Port-au-Prince, Haiti' has been provisionally accepted for publication in PLOS Mental Health.

Before your manuscript can be formally accepted you will need to complete some formatting changes, which you will receive in a follow up email. A member of our team will be in touch with a set of requests.

Please note that your manuscript will not be scheduled for publication until you have made the required changes, so a swift response is appreciated.

IMPORTANT: The editorial review process is now complete. PLOS will only permit corrections to spelling, formatting or significant scientific errors from this point onwards. Requests for major changes, or any which affect the scientific understanding of your work, will cause delays to the publication date of your manuscript.

If your institution or institutions have a press office, please notify them about your upcoming paper to help maximize its impact. If they'll be preparing press materials, please inform our press team as soon as possible -- no later than 48 hours after receiving the formal acceptance. Your manuscript will remain under strict press embargo until 2 pm Eastern Time on the date of publication. For more information, please contact mentalhealth@plos.org.

Thank you again for supporting Open Access publishing; we are looking forward to publishing your work in PLOS Mental Health.

Best regards,

Karli Montague-Cardoso

Staff Editor

PLOS Mental Health

***********************************************************

Reviewer Comments (if any, and for reference):
